# Improved negative selection protocol for *Plasmodium berghei *in the rodent malarial model

**DOI:** 10.1186/1475-2875-11-103

**Published:** 2012-03-31

**Authors:** Rachael Y Orr, Nisha Philip, Andrew P Waters

**Affiliations:** 1 Wellcome Trust Centre for Molecular Parasitology, Institute of Infection, Immunity and Inflammation, University of Glasgow, Glasgow, UK

**Keywords:** *Plasmodium berghei*, Negative selection, Transfection, Selectable marker, Malaria

## Abstract

An improved methodology is presented here for transgenic *Plasmodium berghei *lines that express the negative selectable marker yFCU (a bifunctional protein that combines yeast cytosine deaminase and uridyl phosphoribosyl transferase (UPRT)) and substitutes delivery of selection drug 5-fluorocytosine (5FC) by intraperitoneal injection for administration via the drinking water of the mice. The improved methodology is shown to be as effective, less labour-intensive, reduces animal handling and animal numbers required for successful selection thereby contributing to two of the "three Rs" of animal experimentation, namely refinement and reduction.

## Background

Reverse genetics in malaria parasites has been possible since the early to mid-90s [1-3] and it is now established for a number of *Plasmodium *species that infect a variety of hosts including human (*Plasmodium falciparum*), rodent (*Plasmodium berghei, Plasmodium yoelii *[4] and *Plasmodium chabaudi *[5]) primate (*Plasmodium knowlesi *and *Plasmodium cynomolgi*) as well as avian (*Plasmodium gallinaceum*). Typically genetically transformed parasites are positively selected in the proliferating asexual blood stages by their expression of a marker protein that confers resistance to a common antibiotic and in some cases specifically an anti-malarial drug. The number of selectable markers available for positive selection is limited, where currently five are available for use with parasites that can be cultured *in vitro *for long periods (i.e. *P. falciparum, P. knowlesi *and *P. cynomolgi*). However, selection in rodent malaria parasites may only be carried out *in vivo *as they cannot be cultured long term *in vitro*, reducing the number of positive selectable markers available to three: *Toxoplasma *DHFR-thymidylate synthase (tgDHFR-TS) and *P. berghei *DHFR-thymidylate synthase (pbDHFR-TS), which impart resistance to pyrimethamine and, human dihydrofolate reductase (hDHFR) which confers resistance to both pyrimethamine and WR99210. Therefore, there are only two positive selection regimes available when working with the rodent malarial model since both Apicomplexa possess genes producing DHFR-TS which confers resistance to the same anti-malarial drug, pyrimethamine. Although sequential genetic modification on *P. berghei *using these markers in the strict order of DHFR-TS selection with pyrimethamine followed by hDHFR selection with WR99210 [6] is possible, the number of manipulations is still limited to two. Therefore, an alternative strategy was developed using a negative selection procedure to recycle the both the positive and negative selectable markers, which could then be used multiple times on the same parasite line [7].

Negative selection in reverse genetics typically selects for the loss of a marker in the presence of a suicide substrate that is metabolized by the marker to create a substance that is toxic within the cell under selection. Thymidine kinase is the most well used negative selectable marker and metabolizes either ganciclovir (to ganciclovir triphosphate) or acyclovir (to acyclo-guanosine monophosphate - acyclo-GMP) which compete with GTP for incorporation into DNA resulting in termination of elongation which is lethal to the cell.

**Figure 1:**
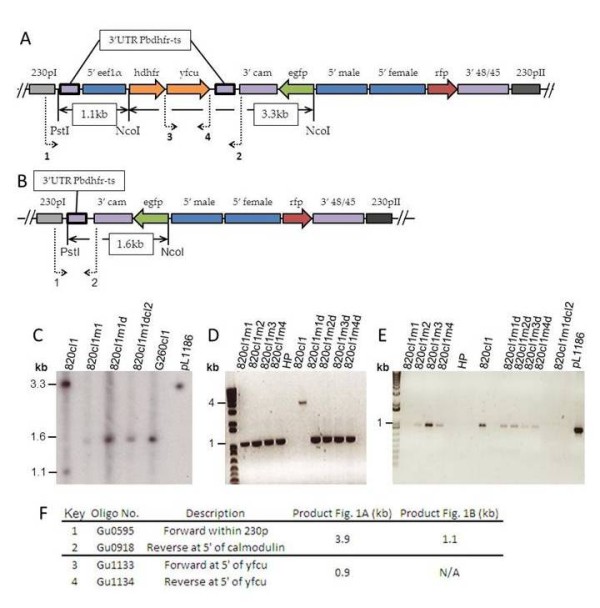
**Negative selection with 5 fluorocytosine a comparison of IP with newly refined oral delivery**. A. Integration locus *230p *with plasmid pL1186. B. Integration locus *230p *post negative selection. C. Southern analysis of negative selection restriction digest. D. PCR of full selection cassette. E. PCR of selectable marker yFCU. F. Primer details. Negative selection with 5 fluorocytosine (5FC) via IP 10 mg/day over 4 days (820cl1m1-m4) and drinking water at 0.5 mg/ml (820cl1m1d-m2d) and 1.5 mg/ml (820cl1m3d-4d) (A) Schematic representation of integration locus *230p *of pL1186 used to create the 820cl1 line at *pb230p *region after positive selection of the double crossover integration event with pyrimethamine, restriction digest with *NcoI-PstI *creates 3.3 kb and 1.1 kb fragments in Southern analysis shown here with thin black arrows (see C). The bold boxes indicate the region of the only feasible recombination event (3'pbdhfr/ts) through negative selection. Bold dotted arrows display the primers 1 & 2 used in diagnostic PCRs to amplify the whole cassette at 3.9 kb (see D & F) and 3 & 4 to amplify yFCU the selectable marker for negative selection of recombinants at 0.9 kb (see E & F). (B) Schematic of gene locus after negative selection with 5 Fluorocytosine (5FC), box in bold represents the footprint left from recombination event after negative selection. Restriciton digest with *NcoI-PstI *results in 1.6 kb band shown by thin arrows. Bold dotted arrows show expected PCR fragment with primers 1 & 2 producing a 1.1 kb band (see F). (C) Southern blot analysis of restriction digest on 10 μg of genomic DNA and 5 ng of plasmid pL1186 with *NcoI *and *PstI *using 3'pbdhrf/ts as a hybridisation probe (bold boxes in Figure 1A and B). Expected band size for negatively selected is 1.6 kb (820cl1m1, 820cl1m1d, 820cl1m1dcl2 and G260cl1) and pre-negative selection is 3.3 kb (820cl1 and pL1186) and 1.1 kb (820cl1). (D) PCR of full selection cassette with primers 1 & 2 (see 1E) to amplify a region of 3.9 kb before selection (820cl1) and 1.1 kb after selection (820cl1m1-4 and 8320cl1m1d-4d) negative control is HP wt with no band. (E) PCR of yFCU negative selectable marker with primers 3 & 4 (see 1E), band of 0.9 kb shows that yFCU is present. Positive control is 820cl1 and pL1186, negative control is HP wt lacking selectable markers. (F) Detailed information of primers used in this study.

An alternative negative selectable marker that has been shown to work well in malaria parasites is a bifunctional protein (yFCU) [7,8] that combines yeast cytosine deaminase and uridyl phosphoribosyl transferase (UPRT): yFCU confers sensitivity to the drug 5-fluorocytosine (5FC) which is metabolized to generate 5' fluorocytosine triphosphate (5FCTP), a lethal analogue of CTP. Therefore, it is possible with a transgenic *P. berghei *line that expresses yFCU to recycle the limited number of positive selectable markers via negative selection and homologous recombination (see Figure 1). The resulting drug sensitive parasite line lacking selectable marker genes may then be genetically manipulated a second time. This recycling event of the selectable marker can be exploited for use on multiple occasions, enabling introduction of transgenes that complement the loss of gene function in knock-outs as confirmation of phenotype.

The procedure that was previously developed for negative selection using 5FC required the administration IP of 1 g/kg 5FC once each day for four days. A typical selection procedure will use five mice and the parasite population that survives generally contains a mixture of parasites that have been genuinely selected and lack the negative selectable marker and parental parasites that have been unaffected by the selection process. Previously, Braks *et al *demonstrated those unaffected by the selection process were due to either no longer possessing the *yfcu *gene anymore or the entire locus being restored. The post-selection populations are then monitored by PCR or Southern blot analysis to determine the ratio of parental to recombined and the population with the most advantageous ratio is used for cloning by limiting dilution. A total of 16 mice are used for this process and under some European regulations mice receiving IP injections must be subjected to light anaesthesia. Of this 16, the negative selection requires five mice. Regulations are being tightened in many countries resulting in new techniques being investigated to reduce animal numbers (personal communications), until these are established the refinement of existing techniques is the only route to conform to governing bodies specifications.

A protocol is reported here that permits the negative selection of *P. berghei *parasites expressing yFCU through the administration of 5FC via the drinking water of the animals, thereby obviating the need for injections and anaesthesia. The drug need only be changed after four days reducing labour and the selected populations are sufficiently pure to allow subsequent cloning permitting the use of only two mice for the initial selection, reducing animal use by one and a half fold. This refinement to the administration of selection drugs has been seen before with pyrimethamine. [9]

## Methods

### Animals and reagents

TO (Theiler's original) or NIH Swiss outbred mouse, female, 20-30 g, aged 6 weeks. 5FC (5-fluorocytosine) (Sigma-Aldrich): 0.5 mg/ml and 1.5 mg/ml in drinking water. All animal procedures were carried out according to Home Office regulations and protocols were approved by the University of Glasgow Ethics Committee. Animals were infected by IP injection with 0.1 ml of a thawed suspension of cryopreserved infected erythrocytes or via passage intraperitoneally.

Negative selection was carried out following either the protocol outlined here using administration of 5FC in drinking water or it was performed according to previously published procedures [7], which is available at the Leiden Malaria Research Group Sharepoint site [9].

### Protocol

This protocol is based upon the use of outbred mice weighing 20-30 g, presuming that the animal consumes, as was observed, 5-10 mls of drinking water daily, to receive an equivalent dose of 80-750 mg/kg/day of 5FC. In a recent human study, 100 mg/kg/day orally was found to be safe over a course of two weeks this was used as a basis for the lower dose [10]. The existing IP delivery method using 1 g/kg/day was considered as a guide for the higher dose in this experiment. Before commencing this study, other drug qualities were noted such as, the half-life which is shown as 3-5 hours before excretion in the urine of a healthy individual [11] and the bioavailability is 76-89% [12]. Previously negative selection was carried out over four days during which 10 mg of 5FC was intraperitoneally injected every day. The 5FC stock was supposed to be at 20 mg/ml in 0.9% NaCl solution, a concentration found past the limit of solubility of the compound, therefore, 1 ml of 10 mg/ml was used. However, as in water it was possible to achieve a 15 mg/ml concentration of 5FC, bulk solutions of lower concentration to be used for administration in drinking water could be easily prepared.

The reporter line 820cl1m1cl1 [RMgm-164] (standard nomenclature used in this field is 'cl' for clone and 'm' for mouse indicating the line has undergone negative selection) was created for sex specific expression of fluorescent markers, *rfp *is female specific expression driven by the *lccl *promoter and male specific *egfp *is driven by the *heavy dynein chain *promoter [13]. For these studies, the parental stock 820cl1 of this reporter line prior to negative selection was used to compare delivery methods. A TO or NIH Swiss outbred mouse weighing 25 g was infected by IP injection on day 0, typically a Friday, with 0.1 ml of a thawed suspension of cryopreserved 820cl1 frozen at a parasitaemia of 3-5% infected erythrocytes. Giemsa-stained thin blood films were observed daily to monitor the parasitaemia. Upon reaching 0.5-3% parasitaemia, a passage was performed into four mice. It has been established locally and determined empirically that an IP injection of 50 μl of cardiac blood at 0.2-1% parasitaemia mixed with 0.2 mls of PBS produces a parasitaemia of 0.2-1.5% after 48 hours, typically a Wednesday. Five hundred microlitres of cardiac blood at parasitaemia 0.5-5%, administered IP would result in the desired infection levels in 24 hours to proceed with negative selection treatment.

The pro-drug 5FC was prepared to concentrations of 0.5 mg/ml and 1.5 mg/ml using vigorous shaking to dissolve the crystals. This drug can be further encouraged into solution by using pre-warmed tap water and a vortex. Drinking water with 5FC was applied in opaque or darkened bottles, as the drug is light sensitive, at a parasitaemia of ≥ 0.1%. The infection levels dropped to zero after 48 hours, in both the 0.5 mg/ml (m1d - 2d) and 1.5 mg/ml (m3d - 4d) dosage sets. Parasites were detected again 48 hours post clearance and, the mice were sacrificed at ≥ 3% parasitaemia to harvest blood for cryopreservation and preparation of leucocyte and erythrocyte free pellets. The drugged water was renewed after 4 days. Once selection was established in the drinking water sets, 820cl1m1d was chosen to produce a clonal line named 820cl1m1dcl2. This positive/negative selectable marker free line was tested for further genetic manipulation by deleting the PBANKA_120440 gene (G260cl1) using a DXO vector pL0035 [7,9]which contains positive and negative selectable markers. The pellets recovered from all the above-mentioned lines were then processed for genomic DNA extraction to perform comparative diagnostic PCRs and Southern analyses alongside the untreated control (820cl1).

### Genotype analysis of parasites post negative selection

Diagnostic PCRs and Southern analysis of restriction digests were performed according to standard methods on the IP control group, the drinking water group, the original 820cl1 pre-negative selection parasite stock and the pL1186 construct used to create 820cl1 [13] (Figure 1). Recombination of the 3'*pbdhfr-ts *homology regions removing the selectable cassette hence verifying that negative selection occurred was demonstrated by PCR amplification of 30 cycles with the external primer pair GU0595 CCgggcccATGACATCATTTATAAATCATG and GU0918 GTAAAGGGTTAATTCTTATATGGTCG.

Further evidence of removal of the selection cassette was confirmed by PCR using primers GU1133 GTGACAGGGGGAATG and GU1134 GATAGCACTACCACCGG to amplify the *yfcu *selectable marker. Southern analysis was completed on genomic DNA of one member from each group, an IP control (820cl1m1), drinking water (820cl1m1d), cloned after selection using drinking water (820cl1m1dcl2), and a line to demonstrate positive selection of a second transfection on the 820cl1m1dcl2 background (G260cl1) and plasmid DNA pL1186. Ten micrograms of DNA was digested with *NcoI *and *PstI *[13] overnight at 37°C and subsequently loaded onto a 1.2% agarose gel stained with Sybr safe. The resulting blot was then hybridized with the 3'pbdhfr-ts probe amplified from pL1186 DNA template with primer pair LUMC692 CTTATATATTTATACCAATTG and LUMC693 GTTTTTTTTTAATTTTTCAAC.

## Results

### Refinement to negative selection of recombinant parasites *in vivo*

Negative selection pressure on mutant parasites with a disrupted gene using oral administration of the pro-drug 5FC was verified to be as efficient as the existing method applied intraperitoneally. The predicted homologous recombination event (Figure 1A/B) resulting from effective negative selection is evident by Southern analysis (Figure 1C) with only the original 820cl1 line (3.3 kb and 1.1 kb) and pL1186 plasmid (3.3 kb) showing any presence of the selection cassette (Figure 1A/B). The negatively selected 820cl1m1, 820cl1m1d and G260cl1 all display the 1.6 kb band expected after homologous recombination of 3'pbdhfr/ts demonstrating that this line is amenable to additional manipulations subsequent to the selection process. The parasite line G260cl1 was created through transfection of 820cl1m1dcl2 with a PBANKA_120440 KO construct introducing the pL0035 plasmid which possesses both positive and negative selectable markers, resulting in a line that was positively selected for a second time and subsequently cloned.

More sensitive diagnostic PCRs with 60 ng of DNA per reaction as standard was quantified by NanoDrop 1000 from Thermo Scientific (DNA can be quantified spectrophotometrically by other means) have shown that the selection cassette is only present in 820cl1 parasite line at 3.9 kb whilst in all of the standard IP control group (m1 - 4) and orally administered mice (m1d & 2d on 0.5 mg/ml 5FC and m3d & 4d on 1.5 mg/ml 5FC) the presence of a smaller 1.1. kb band confirms the absence of the selection cassette (Figure 1D). To supplement this finding, additional PCRs were carried out on sub-sections of the cassette, all of which given the non-quantitative nature of PCR were present at apparently well-reduced levels in both the IP control group and oral group in comparison with the untreated 820cl1 line. Further cloning by serial limiting dilutions resulted in a pure population without the selection cassette yielding the 820cl1m1dcl2 line. Levels of the *yfcu *PCR product varied greatly amongst the IP control group from almost zero in 820cl1m1 to as much as was observed in the selection cassette positive line 820cl1 in 820cl1m3. Conversely the drinking water group consistently had lower levels of *yfcu *than 820cl1. As expected, the oral group apparently responded in a dose dependent manner with 820cl1m3d and 820cl1m4d, which were on the higher dose of 1.5 mg/ml had less *yfcu *present than the 820cl1m1d and 820cl1m2d 0.5 mg/ml pair (Figure 1E).

## Discussion

Evidence suggests that the adapted method of orally administered 5FC for negative selection is just as effective as the existing IP method currently utilized. Furthermore, the modified technique has the additional benefits of reducing animal numbers required and refining their treatment by removing the need for injectables in this protocol. Moreover there are financial gains made by reduction in cost for the animals, their housing, consumables, drug, and labour. The procedure may be carried out in the UK by personnel who do not possess a personal Home Office animal license, although an amended list of designated duties is added to the laboratory license. Regional policies may differ. A comparison between two doses for oral administration was drawn in this experiment with the same successful end result (Figure 1D). Currently we use 1 mg/ml 5FC, which sits between the two doses of 0.5 mg/ml and 1.5 mg/ml. It would be recommended that if no reduction in parasitaemia is observed after 24 hours that the dose can be increased to 1.5 mg/ml to ensure full clearance of parasites. A mouse from the lower dose (820cl1m1d) was chosen to clone (820cl1m1dcl2) demonstrating that even at lower doses this method is reliable. It has been observed since on each occasion negative selection using doses ranging between 0.5 to 1.5 mg/ml in drinking water that a homologous recombination event has been successful in every mouse.

The method above describes using five mice in total for selection, one as a donor to propagate the parasites for passage and four to carry out the negative selection. The protocol has been modified further with personal observations that by using two mice instead of four for the selection, they can be infected with 50 μl from one cryopreserved stabilate on day 0 removing the need for three mice. The recombined product validating successful negative selection has been achieved on every occasion. This would mean that in comparison to the previous method a negative selection and cloning procedure could be concluded with as little as 13 mice. In all experiments where negative selection pressure has been administered through drinking water-based delivery of 5FC it was possible to clone parasite lines by limiting dilution that had undergone the anticipated recombination event. No false positive lines were generated indicating that oral administration of 5FC produces sufficiently pure recombined populations of parasites for further use.

## Conclusion

The modified negative selection method described here is shown to work successfully at a range of doses without bringing harm to the animal. This protocol fulfils two of the "three R's", refinement and reduction appeasing the requirements of governing bodies monitoring animal usage. Moreover it reduces the animal numbers required hence cutting housing, reagents, consumables, and labour costs as well as time to produce reliable and consistent results that are easily achieved. *In principle this approach is applicable to transfection studies with all rodent malaria parasite models*.

## Abbreviations

5FC: 5 fluorocytosine; 5FCTP: 5' fluorocytosine triphosphate; DHFR: Human dihydrofolate reductase; DHFR-TS: Toxoplasma DHFR-thymidylate synthase.

## Competing interests

The authors declare that they have no competing interests.

## Authors' contributions

APW conceived of the study, participated in its design and co-ordination and helped draft the manuscript. RYO participated in the design of this study, carried out drug selection and molecular genetic studies and drafted the manuscript. NP generated a transgenic line and helped to draft the manuscript. All authors read and approved the final manuscript.
